# Does Smart City Construction Reduce Haze Pollution?

**DOI:** 10.3390/ijerph192416421

**Published:** 2022-12-07

**Authors:** Li Wang, Qian Xie, Fei Xue, Zongxin Li

**Affiliations:** 1 School of Economics & Management, Northwest University, Xi’an 710127, China; 2 Faculty of applied economics, University of Chinese Academy of Social Sciences, Beijing 102488, China

**Keywords:** smart city, haze pollution, multi-time difference-in-differences, benefit analysis

## Abstract

Smart city construction plays an important role in environmental governance and public health. Based on the panel data of 216 prefecture-level cities across China during the period 2009–2018, this study uses the multi-time difference-in-differences method to evaluate the haze reduction effect of smart city construction. The estimated results demonstrate that the construction of smart cities can reduce haze pollution in pilot cities significantly. The main conduction mechanisms are the technical effects and the environmental regulatory effects that promote the reduction of corporate emissions. The heterogeneity analyses show that the haze reduction effect of smart city construction is more evident in southern cities, inland cities and resource-efficient cities. In addition, the benefit analyses show that smart cities can reduce the carbon emission intensity and promote economic growth. These results provide empirical support for accelerating the construction of a new type of smart city and building a new type of people-oriented urbanization.

## 1. Introduction

Presently, urbanization in China has entered a period of healthy and sustainable high-quality development; such that the 14th Five-Year Plan proposes to follow the new concept and trend of urban development, improve the quality of urbanization development, and enable more people to enjoy a higher quality of urban life [1]. However, environmental pollution, traffic congestion, housing tension, and other “urban diseases” are still facing shortcomings in governance, especially environmental pollution problems such as haze, which seriously affect the quality of healthy life. After several years of efforts, governments at all levels have taken a variety of measures to “iron fist haze” [2]. Although air quality has also continued to improve, much can still be accomplished regarding air pollution prevention. Report on the State of the Ecology and Environment in China 2019 shows that in 2019, the ambient air quality exceeded the standard in 180 of the 337 prefecture-level and above cities in the country, with the average percentage of days exceeding the standard reaching 18%; while the number of days containing PM_2.5_ in the air, the main pollutant, accounted for 78.8% of the heavily polluted days and above [2]. To enter a new stage of green development, in contrast with other special policy measures for haze pollution management, pollution prevention by enhancing the level of urban smartness which refers to the city’s governance and service capacity improved according to building smart cities in the process of new urbanization construction will become essential.

Smart city construction provides a new way to solve various urban diseases caused by rapid industrialization and urban expansion and promote sustainable urban development. In 1992, Singapore took the lead in proposing the “Smart Island Project” as a prototype of the “Smart City” concept [3]. In 2008, IBM proposed the strategy of “Smart Earth” [4], which officially set off the boom of smart city construction in various countries. The essence of smart cities lies in the high degree of integration of information technology and urbanization, using advanced information technology such as the Internet of Things, cloud computing and big data to lead a new round of technological revolution and industrial change [4]. Related concepts have been proposed one after another, such as “Smart Cities and Communities Innovation Partnership” in the EU, “Digital Britain” action plan in the UK, and “U-City” smart city in South Korea [5,6,7]. This paper argues that the core concepts of smart city construction include wisdom with regard technology, industry, service, and management that serve the high-quality development of urbanization; it further involves new-generation information technology such as the Internet of Things, cloud computing, big data, and spatial geographic information integration.

The Chinese government is also keeping pace with advanced city construction around the world. The Department of Information Industry of Guangdong Province signed a memorandum of cooperation with IBM in 2009 to strengthen the field of information technology and information services to promote the construction of smart cities, marking the official opening of the construction of smart cities in China, thereby opening the practical exploration of the construction of smart cities with Chinese characteristics [8]. The Ministry of Housing and Construction issued the “Notice on National Smart City Pilot Work” and implemented the first batch of smart city pilots in more than 90 cities at the end of 2012 and “Guidance on Promoting the Healthy Development of Smart Cities” in 2014 [9,10]. The notice states smart cities digitize various resources and facilities by means of information technology, thus realizing intelligent planning and intelligent management of cities, improving the quality of life of residents, promoting a higher level of sustainable development of cities, and providing new impetus for economic transformation [9]. Additionally, the guideline for the construction of smart cities is to follow the general requirements of taking the new road of intensive, intelligent, green, and low-carbon urbanization; play the decisive role of the market in resource allocation; strengthen and improve government guidance; coordinate material, information, and intellectual resources; strengthen the intelligent construction of urban management and service systems; effectively improve the comprehensive carrying capacity of cities and residents’ happiness; and promote the overall improvement of the quality and level of urbanization development [10]. In August 2013, the Ministry of Housing and Construction added 103 cities, including the Beijing Economic and Technological Development Zone, as pilot smart cities [11]. In 2014, the Ministry of Housing and Construction and the Ministry of Science and Technology listed 97 cities, including Mentougou District in Beijing, as pilot cities [12]. In 2015, the Central Internet Information Office put forward a new concept called “new type of smart city”, marking a new stage in the construction of smart cities in China [13]. In 2019, the National Development and Reform Commission listed the construction of a “new type of smart city” as a key task for new urbanization construction [14]. The COVID-19 outbreak in 2020, health codes, remote online consultation, and other wisdom applications have extended the connotation and extension of the new smart city, and smart city construction entered an explosive growth period. By the end of 2020, the number of smart city pilots announced by the Ministry of Housing and Construction reached 290 [15].

However, the question whether or not such new urbanization measures are conducive to improving environmental quality and promoting green and sustainable urban development urgently needs to be answered. Thus, this paper focuses on the impact of smart city construction on haze pollution by taking the smart city pilot policy as an entry point. Does the construction of smart cities help reduce the level of haze pollution in pilot cities? If the answer is yes, what are the mechanisms that influence haze pollution? Are there regional heterogeneities in their haze pollution control effects? Can smart city construction achieve collaborative governance of haze and carbon emissions under the dual challenges of air pollution and climate change? Are the governance effects achieved at the expense of economic benefits? All these issues deserve to be discussed further.

Existing literature reviews mainly focus on two aspects: the first is about the influencing factors of haze pollution. Many scholars have examined the factors influencing haze pollution from multiple perspectives and have achieved many results. Fiscal decentralization, industrial agglomeration, energy prices, transportation infrastructure, and urbanization are all important factors that influence haze pollution [16,17,18,19,20,21]. Highly relevant to this study is the fact that some scholars have examined the haze-control effects of a range of policies in recent years. For example, Wang and Shi and Song examined whether or not low-carbon city construction could mitigate air pollution [22,23]. The study by Luo and Li demonstrates that the “Atmospheric 10” policy is beneficial in reducing haze pollution levels in some regions [24]. The second is smart city-related research. Since the concept of a smart city has been proposed, much research has been conducted around its essential connotation, realization path, and development model, as well as a comprehensive evaluation of its development [25,26,27,28]. Meanwhile, some scholars have also focused on assessing the policy effects of smart city construction, such as the impact on industrial structure, urban innovation, corporate total factor productivity, and energy efficiency [29,30,31,32,33]. In contrast with the aforementioned literature, which focuses on economic indicators, Shi et al. and Cui and Chen estimated the impact of smart city construction on urban industrial pollution using the traditional difference-in-difference (DID) method and the multi-time DID method, respectively, and showed that smart city construction reduced urban wastewater and waste gas emissions [34,35]. Recently, Feng and Hu found the spatial spillover effect of smart city policies based on the result that smart city policies reduced urban haze pollution in pilot cities [36]. The above literature provides a multifaceted assessment of the Chinese government’s actions, reflecting the role of smart city construction.

By combining the above literature, many useful results have been obtained concerning the research on the policy effects of smart city construction, but there are a few studies in the literature directly discussing the effects of smart city construction on haze pollution, which provides a research opportunity for this paper. As such, this study takes the panel data of 216 prefecture-level cities in China from 2009 to 2018 as research sample and uses the multi-time difference-in-differences (DID) method to examine the impact of smart city construction on haze pollution. The contributions of this study include: (1) Existing studies focus on the economic effects of smart cities, where limited attention is paid to environmental effects, especially haze management effects. This study contributes to the literature by focusing on the effect of smart city construction on haze reduction, which may improve urban governance and residents’ happiness. This study contributes to the literature by focusing on the effect of smart city construction on haze reduction, which may improve urban governance and residents’ happiness. (2) In terms of data processing and method selection, this paper takes the raster PM_2.5_ from Dalhousie University and converts it into panel data of prefecture-level municipalities, which overcomes the barrier of discontinuity in the available data and overcomes the possible endogeneity problems of using the DID method. (3) Based on mechanism analysis, this study not only explores the haze reduction mechanism of smart city construction, but also finds that smart city construction can reduce carbon emission intensity and promote economic growth in pilot cities, which provides a new path to effectively solve urban diseases and promote high-quality urban development.

## 2. Theoretical Hypothesis

Fundamentally, smart city construction is a systematic and innovative project of reform, which is the centralized embodiment of scientific and technological innovation, organizational innovation, and resource-allocation innovation. In theory, this innovation effect is primarily reflected in haze control through smart environmental protection, technology, and haze-control technology, as well as smart transportation and smart buildings.

First, smart environmental protection requires a total of six modern information technologies, including comprehensive use of the Internet of Things, cloud computing, intelligent GIS, integrated remote-sensing monitoring, massive data mining, and environmental model simulation technology, to provide comprehensive solutions for environmental-quality-monitoring systems, environmental early warning and forecasting systems, environmental emergency management systems, and other areas of environmental protection. This enables environmental policymakers and air pollution controllers to accurately grasp trends in environmental quality and thus provide data support for haze-reduction actions. Second, technological innovation is an important means for haze management. In the process of smart city construction, the green technology for haze control has been fully utilized, such as the “multi-scale spraying and haze removal technology” used in Chengdu City and the haze removal tower built in Xi’an City. Third, the development of intelligent transportation in the construction of smart cities also indirectly contributes to haze control. The planning of the shortest travel path or the best route in the intelligent transportation system helps the public to choose the best driving route, thus effectively alleviating traffic congestion and reducing pollutant emissions. Fourth, smart city construction is conducive to the promotion of smart buildings, which use energy-saving materials and technologies in the planning, design, construction, and use process, thereby alleviating haze pollution.

Based on the above understanding, the first hypothesis is proposed as follows:

**Hypothesis** **1.** *Smart city construction is beneficial for reducing the level of haze pollution in pilot cities.*

At the same time, possible transmission routes are discussed as follows:

The first is the technology effect. Technological innovation is an important driving force for green development and important support for fighting the battle of pollution prevention and control, thereby promoting ecological civilization construction and high-quality development. The construction of smart cities is conducive to the promotion of research and development of green technology and its application, and the technological progress it offers can effectively improve capabilities for pollution prevention and control. For example, the intelligent environmental protection big data system can observe and analyze the air pollution situation in real time, such that environmental managers can use the system to effectively manage pollution sources and polluting industries, thereby reducing haze pollution levels.

Second are the structural effects. Smart city construction reduces haze pollution by promoting development of smart city industry and upgrading of industrial structure. Unlike the traditional development model, the smart city is a new development model supported by a new generation of information technology, which has generated a huge demand for emerging wisdom technologies and promoted the formation and development of the smart city industry and its associated industries. The development of the smart city industry is conducive to accelerating the upgrading of urban industrial structures and the construction of a modern urban industrial system, thus creating a smart city ecological industrial chain. Among them, characteristics of low pollution, low energy consumption, low emissions, high-tech, and high industrial added value of the smart city industry will reduce haze pollution.

Third is the environmental regulation effect. Environmental regulation, as an important element of social regulation, is an important tool for improving haze pollution. Distinct from general pollution control policies, smart cities can strengthen environmental regulations by strengthening environmental monitoring systems, thereby reducing haze pollution. On the one hand, the integrated environmental monitoring system of smart cities can be used to monitor the pollutant emission status of enterprises in an all-around and all-time manner and restrict the illegal emission behavior of relevant enterprises. On the other hand, in the process of smart city construction, the public has access to more comprehensive pollution emission data, which enhances the public awareness regarding environmental protection, thereby enhancing the intensity of public-participation-based environmental regulation.

Considering the above factors, we present the second hypothesis:

**Hypothesis** **2.** *Smart city construction affects haze pollution levels in three ways, through technological, structural, and environmental regulation effects.*

## 3. Study Design

### 3.1. Model Setting

In 2012, 2013, and 2014, construction of smart cities in China was introduced in batches through promotion of pilot projects which were expected to inevitably ensure operational experience and help form long-term operational mechanisms. However, the overall knowledge is still in its infancy.

The smart city pilot policy in China is a good “quasi-natural experiment” in that the selection of the pilot is not randomly assigned. Therefore, this paper uses the multi-time difference-in-differences method, which is one of the methods in the quasi-natural experiment, to estimate its impact on haze control. Research subjects were identified by the smart city pilot list announced by the Ministry of Housing and Construction, and the prefecture-level cities that have implemented the smart city pilot were selected as the test group samples, while the remaining prefecture-level cities that were not approved for smart city construction were considered as the control group sample. A prefecture-level city is one of China’s administrative divisions, with the same administrative status as a region, autonomous prefecture, or union, under the jurisdiction of a province or autonomous region. The following criterion were used to construct the sample: (1) Exclude prefecture-level cities that are not fully piloted; that is, those that implement smart city construction only in certain districts or counties within the city. The reason is that some of the pilot units of smart cities are implemented in county-level areas, e.g., Chaoyang District, Beijing, and Heihe County, Jinzhou City. Setting these cities with partial regional implementation of pilots as pilot cities would lead to an overestimation of the estimated results. (2) Exclude newly established prefecture-level cities within the study interval (2009 to 2018), such as Bijie and Tongren. During the study period of this paper’s sample, these cities experienced administrative divisional adjustments. For example, in 2011, the State Council approved the abolition of the Bijie district to establish the prefecture-level Bijie city; on 22 October 2011, the Tongren district was abolished to establish the prefecture-level Tongren city. Additionally, there is a large number of missing statistics for these cities before their elevation to non-prefecture-level cities. (3) Exclude prefecture-level cities with serious data deficiencies, such as Lhasa. Finally, the dataset of 91 cities in the experimental group and 125 in the control group was obtained. Among the samples in the experimental group, there were 35, 33, and 23 prefecture-level cities that implemented smart city construction in 2012, 2013, and 2014, respectively. The model was set as follows:

(1) $$Y_{it}=\alpha_{0}+\alpha_{1}Smart_{it}+\alpha_{2}X_{it}+\mu_{i}+v_{t}+\varepsilon_{it}$$

where $Y_{it}$ is the explanatory variable indicating the level of haze pollution in city i in year t. $X_{it}$ is a control variable indicating the variables that may affect the level of haze pollution. $v_{t}$ represents the time fixed effect, $\mu_{i}$ represents the individual fixed effect for each city, and $\varepsilon_{it}$ is the random error term. $Smart_{it}$ is the core explanatory variable, indicating whether city i is a smart city pilot in year t. If so, it takes the value of one; otherwise, it takes the value of zero. $\alpha_{1}$ is the main coefficient of interest in this study, which measures the policy effect of the impact of smart city construction on haze pollution levels, and if it is significantly negative, it indicates that the smart city pilot policy suppresses haze pollution.

### 3.2. Variable Definition and Data Description

The study herein focuses on assessing the haze control effect of smart city construction while considering the possible endogeneity problems caused by omitted variables, and following Sun et al., a series of control variables are also included [37]. The variables are set as follows.

(1) Haze pollution level Following Chen and Xiao, this study used annual average PM_2.5_ concentrations to measure the haze pollution level [38]. Since 2013, the Ministry of Ecology and Environment began to publish PM_10_, PM_2.5_, and other haze-related data, which means that the sample interval 2009–2018 contains incomplete data. Therefore, the continuous PM_2.5_ data published by the Atmospheric Composition Analysis Group of Dalhousie University in Canada were used in this study from 2000 to 2018 [39]. However, the original data are in raster form, and ArcGIS software was used to parse it into prefecture-level city panel data that can be used in the model.
(2) CO_2_ emission and CO_2_ emission intensity. The DMSP and VIIRS night-light data were used to estimate the CO_2_ emissions and the CO_2_ emission intensity of the sample cities, which were, respectively, used as explained variables for regression in the other benefit analysis section. The data come from the study of Chen et al. [40].
(3) Smart City pilot policy. Whether a city is a smart city pilot will be measured by a dummy variable. The dummy variables were assigned according to the smart city pilot list announced by the Ministry of Housing and Construction. If a city was identified as a smart city pilot, the dummy variable was assigned a value of 1 for the current year and subsequent years; otherwise, it was assigned a value of 0. The values of this variable were manually compiled.
(4) Control Variables were the level of economic development, expressed as the logarithm of the real GDP per capita of each prefecture-level city, which also incorporates the squared term of the level of economic development to test the existence of the Kuznets curve hypothesis; climate condition, measured by the logarithm of average annual precipitation; government size, measured by the logarithm of government fiscal spending as a share of GDP; the level of financial development, measured by the logarithm of the loan balance of financial institutions as a share of GDP at the end of the year; the level of greening, expressed as the logarithm of green space per capita in prefecture-level municipalities; the level of openness to the outside world, measured by the logarithm of the proportion of the actual use of foreign investment in GDP in that year. Basic data were obtained from the China City Statistical Yearbook and the China Regional Statistical Yearbook for each year. Descriptive statistics of the main variables are shown in Table 1.

## 4. Empirical Results and Analysis

### 4.1. Baseline Model Regression Analysis

Table 2 reports the results of the baseline regression of smart city construction on haze pollution, where column (1) is the regression result without control variables and column (2) is the regression result with control variables. The result reveals that the estimated coefficient of Smart, the smart city pilot, remains significantly negative regardless of whether control variables are included, which indicates that the smart city construction is conducive to promoting haze control and reducing haze pollution levels. Further analysis of the regression results in column (2) shows that the contribution of the smart city pilot to the haze pollution level is −1.27, indicating that the smart city construction led to a decrease of 1.27 micrograms per cubic meter in the annual average PM_2.5_ concentration in the pilot city compared to the control group. In total, the estimated coefficients of the baseline model capture the average treatment effect over seven years, which implies that the smart city pilot policy contributed to a reduction in PM_2.5_ concentrations of about 0.181 ug/m^3^ per year. The annual average PM_2.5_ concentration in the control group cities was 42.6 micrograms per cubic meter. The smart city construction resulted in a 2.98% reduction in the annual average PM_2.5_ concentration in the pilot cities.

The estimated coefficients of the control variables show some interesting results. The estimated coefficients of actual GDP per capita and its squared term are −35.677 and 1.283, respectively, both of which are significantly negative at the 1% level in Table 2. The effect of the logarithm of per capita GDP on PM_2.5_ is changing at 13.906 (the U-shaped inflection point is at 13.906), and the maximum value of the logarithm of per capita GDP in the sample of this paper is 12.655. This indicates that the relationship between economic development and haze pollution is already in the second half of the inverted U-shaped curve, which is inconsistent with the findings of Shao et al. [41]. Climatic condition has a significant negative effect on haze pollution levels; that is, the more precipitation there is, the lower the haze pollution level. The effect of government size on the level of haze pollution is significantly positive; that is, the larger the share of government fiscal expenditure in GDP, the more serious the haze pollution in cities; this may be caused by the process of stimulating economic growth through fiscal expenditure, such that the regulation of polluting industries is indirectly relaxed, leading to increased haze pollution. The level of financial development will enhance haze pollution, which may be due to the slow development of green finance emphasizing environmental friendliness, and the development tends toward the development of industries with fast short-term benefits but may have relatively high pollution, thus indirectly aggravating haze pollution. The greening level has a significant positive effect on haze pollution, and the level of openness to the outside world has a significant inhibitory effect on haze pollution.

### 4.2. Parallel-Trend Tests

A basic condition which must be satisfied for the policy-effect assessment using the DID method is that without the intervention of the smart city construction policy, the urban haze pollution levels in the control and treatment groups should have a common trend of change; that is, a parallel trend assumption needs to be satisfied. Following the method of Beck et al., the dynamic DID model is used to perform a parallel trend test [42]. The specific formula is as follows:

(2) $$Y_{it}=\alpha_{0}+\overset{k=6}{\underset{k=-5,k\neq -1}{\sum }}\beta_{k}D_{i,t_{0}+k}+\delta X_{it}+\upsilon_{t}+\mu_{i}+\varepsilon_{it}$$

where $D_{i,t_{0}+k}$ is a series of dummy variables indicating the *k*th year the smart city pilot policy was implemented. Specifically, *k* < 0 indicates the *k*th year before the pilot implementation; *k* = 0 indicates the year of smart city pilot implementation; *k* > 0, it indicates the *k*th year after pilot implementation. By the end of 2018, the first and last batches of smart city pilots were implemented in 2012 and 2014, respectively, and the sample interval in this study is from 2009 to 2018. As such, this study covered the first five and last six years of pilot implementation. The first year prior to the establishment of the smart city is taken as the base period, so k≠−1 in Equation (2). If $\beta_{k}$ is not significantly different from 0 when *k* < 0, it means that the cities in the control and experimental groups satisfy the parallel trend hypothesis in terms of haze pollution levels.

**Figure 1:**
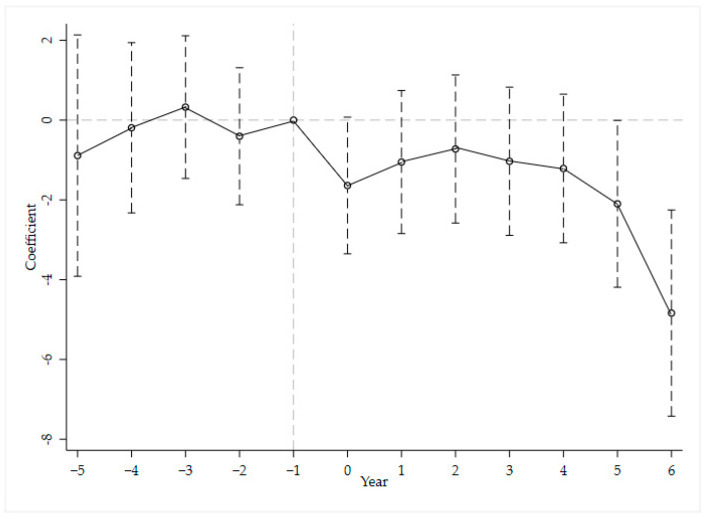
Dynamic effect of smart city policy on PM_2.5_. Note: The solid line in the figure plots the estimated value of the smart city pilot policy, and the dashed line indicates the 95% confidence interval.

Figure 1 illustrates the results of the parallel trend test. $\beta_{k}$ was not significant at the 5% significance level when *k* < 0, and both fluctuate around zero, indicating that the treatment and control groups satisfy the parallel trend hypothesis. When k≥5, the estimated value of $\beta_{k}$ was significantly negative at the 5% significance level; that is, the smart city pilot policy significantly suppresses haze pollution in the 5th and 6th years after implementation, indicating that the haze control effect of smart city construction has a time lag, and the longer the policy is implemented, the stronger the suppression effect on haze pollution.

### 4.3. Placebo Test

**Figure 2:**
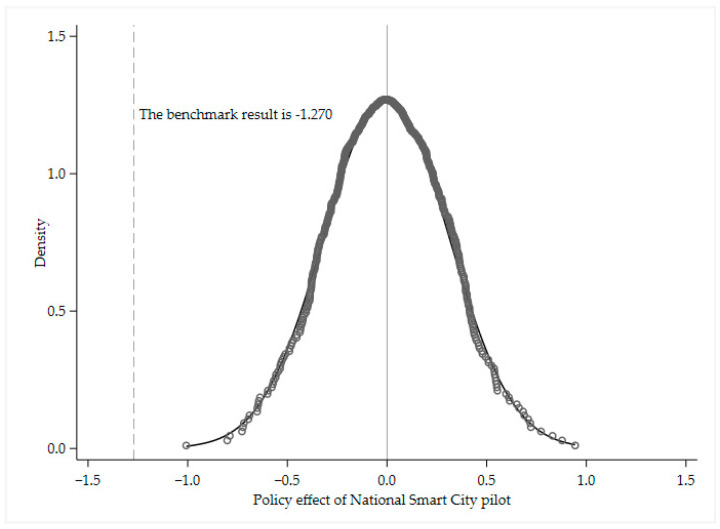
Placebo test results.

The effect of urban characteristics that do not vary over time on haze pollution was controlled by adding the urban fixed effects above, but some of the omitted variables vary over time, which affects the accuracy of the estimation results. Therefore, to test whether the baseline estimation results were affected by omitted variables, such as that in Chetty et al., this paper constructs DID variables based on a randomly generated dummy list of smart city pilots and conducts 500 baseline regressions; the specific regression results and their distributions are shown in Figure 2 [43]. The coefficients obtained based on random sample estimation are distributed around zero, which is far from the baseline regression results, and indicates that the impact of smart city pilot on haze pollution is not disturbed by the omitted variables.

### 4.4. Self-Selection Issues

The most ideal situation using the difference-in-difference method is that pilot and non-pilot cities are randomly selected. In reality, however, the determination of the list of smart city pilots is not random. According to the “Notice on National Smart City Pilot Work” issued by the Ministry of Housing and Construction, the declaration of smart city pilot work requires the inclusion of smart city construction work in the local National Five-Year Plan or relevant special plans, the completion of the preparation of the outline of the smart city development plan, and the existence of a clear funding plan and guaranteed channels. These conditions can reflect the inherent differences between cities, which have an impact on urban haze pollution levels with time trends, leading to bias in the estimation results. Following Lu et al., the stochastic selection problem of pilot cities was mitigated by including, in the benchmark equation, an interaction term between the benchmark factors affecting the selection of pilot cities and the linear trend over time [44]. The specific model is as follows:

(3) $$Y_{it}=\alpha_{0}+\alpha_{1}Smart_{it}+\alpha_{2}X_{it}+Z\times trend+\mu_{i}+v_{t}+\varepsilon_{it}$$

Among them, Z indicates the benchmark factors affecting the determination of the smart city pilot list, including whether it is a provincial capital city, a larger city, and an eastern city, and whether the smart city construction is included in the twelfth Five-Year Plan of Local National Economic and Social Development or relevant special plans and local government financial budgets prior to the pilot implementation. trend represents a linear trend over time. Z×trend depicts the effect of inherent inter-city differences on haze pollution from a linear perspective, and is able to mitigate the estimation bias owing to the non-random selection of the experimental group samples. The corresponding estimation results are reported in column (1) of Table 3, where the coefficient of the smart city pilot is still significantly negative, indicating that the estimation results remain robust after considering the sample self-selection problem.

### 4.5. Other Policy Interference

Other environmental policies pursued during the sample period of this study may have confounded the baseline estimation results. Therefore, this study compiled the large environmental policies implemented since 2009 at the city level, specifically the Low-Carbon-City Pilot Policy implemented in 2010, the Special Emission Limits for Air Pollutants Policy, and the Air Pollution Prevention and Control Action Plan implemented in 2013. Referring to the way the smart city policy variables are set up, cross-terms were constructed based on the corresponding list of cities and the time point of policy implementation, which were added to the baseline regression equation, thus controlling the effects of these three environmental policies on the estimation results. The corresponding estimated coefficients are reported in Column (2) of Table 3. The coefficients do not change notably in significance and magnitude compared to the baseline estimation results, which indicates that the smart city pilot still significantly reduces the haze pollution level after excluding other policy interferences.

### 4.6. Robustness Test

(1) Special samples and outliers were excluded. First, considering that the large-scale haze control actions pioneered in the Beijing–Tianjin–Hebei region since 2013 may interfere with the results, all prefecture-level cities in the Beijing–Tianjin–Hebei region were excluded from the regression, and the regression results are shown in column (1) of Table 4. Second, to avoid the interference of extreme values on the regression results, the explanatory variables were subjected to top and bottom 5% tail reduction and regressed again; the regression results are shown in column (2) of Table 4. The estimated coefficients of the smart city are significantly negative in both of the above treatments, and the magnitude of the coefficients is similar to the benchmark results, which verifies the robustness of the benchmark results.

(2) The explanatory variables were treated with a one-period lag. Considering the lagged effect of smart city construction on haze pollution, a lagged one-period treatment was applied to the smart city variable. To avoid errors in the joint cubic equation, all explanatory variables were lagged by one period, as shown by Shen and Jin [45]. The regression results are reported in column (3) of Table 4, and the conclusions of the baseline estimates in this study still hold.

(3) Following the method in Fan and Tian, time-counterfactual tests were conducted by changing the time of policy implementation [46]. The specific operation was to advance all smart city pilot construction times uniformly for two years, and if the estimated coefficient of the smart city pilot still significantly negative at this time, it indicates that the reduction of haze pollution is derived from other policies or other stochastic factors; if the estimated coefficient of the smart city pilot is not significant, it verifies that the reduction of haze pollution is brought about by the smart city construction. The regression results are shown in column (4) of Table 4. The hypothetical smart city construction pilot variable does not have a significant effect on haze pollution, which indicates that the smart city pilot policy does suppress haze pollution.

## 5. Further Analysis

### 5.1. Mechanism Testing

The results of the above-mentioned types of tests show that the smart city construction significantly reduces the level of haze pollution. Therefore, what are the specific conduction mechanisms? According to the theoretical analysis, the smart city construction may affect haze pollution in three ways: technological innovation effect, industrial structure effect, and environmental regulation effect. Herein, following the method of Baron and Kenny, causal stepwise regression was used to identify the three aforementioned possible transmission pathways [47]. The specific models are as follows:

(4) $$PM2.5_{it}=\alpha_{0}+\alpha_{1}Smart_{it}+\alpha_{2}X_{it}+\mu_{i}+v_{t}+\varepsilon_{it}$$

(5) $$M_{it}=\beta_{0}+\beta_{1}Smart_{it}+\beta_{2}X_{it}+\mu_{i}+v_{t}+\delta_{it}$$

(6) $$PM2.5_{it}=\theta_{0}+\theta_{1}Smart_{it}+\theta_{2}M_{it}+\theta_{3}X_{it}+\mu_{i}+v_{t}+\tau_{it}$$

where $M_{it}$ is the mechanism variable: technological innovation, industrial structure, and environmental regulation, where the technological innovation effect was measured by the number of green patent applications per practitioner. The industrial structure effect was measured using the added value of the secondary sector as a share of GDP. The environmental regulatory effect was measured using industrial SO_2_ emissions for the following reasons. First, SO_2_ pollution is an important factor in haze pollution, and also a key environmental indicator concerned by Chinese governments. China set up acid-rain-control zones or sulfur dioxide pollution-control zones to control sulfur dioxide pollution as early as 1998, and further included sulfur dioxide emissions as a binding indicator in the national economic and social development plan from 2006. Therefore, the changes in SO_2_ emissions could reflect the intensity of environmental regulation by local governments to a certain extent. In addition, the data of sulfur dioxide emissions of each prefecture-level city are published in the China City Statistical Yearbook since 2003, which brings convenience to this study. Second, although the emission of particulates is an important source of PM_2.5_ pollution, no official data on particulate matter emissions at the city level have been published in China to date. As such, the specific mechanism of haze pollution reduction by smart city construction can be tested according to Equations (4)–(6). This is true specifically if $\alpha_{1}$, $\beta_{1}$, and $\theta_{2}$ are all significant, and the absolute value or significance of the coefficient of smart city construction in Equation (5) is reduced compared with the baseline regression, indicating that the transmission mechanism is present.

The results of the mechanism analysis are listed in Table 5. The coefficients of smart cities in columns (1), (2), and (3) were significant when technological innovation was used as the mechanism variable, and the absolute value of the coefficient in column (3) was smaller than that in the baseline regression, indicating that improved green technological innovation in smart city construction can reduce haze pollution. Similarly, the intensity of environmental regulation in smart city construction can influence the level of haze pollution. When the industrial structure was the mechanism variable, the estimated coefficient of industrial structure in column (5) was not significant, indicating that influencing industrial structure in smart city construction does not affect haze pollution. The mechanism analysis concludes that the technological innovation effect and the environmental regulation effect are the principal pathways affecting the level of haze pollution in the smart city pilot policy, whereas the transmission mechanism of industrial structure does not, probably because the smart city construction is still in its infancy, the proportion of emerging industry development is low, and the role of industrial structure adjustment to reduce haze pollution is small. Thus, the conclusion is drawn that Hypothesis 2 is only partially valid.

### 5.2. Heterogeneity Analysis

The average effect of smart city construction on haze pollution was analyzed above. However, the analysis based on the overall sample may have masked the differences that existed between regions. In particular, there are large differences in geographic location, resource endowment, population, and economic agglomeration among regions in China, resulting in very different levels of haze pollution from city to city. To this end, this study further tests whether smart city construction has a heterogeneous effect on haze pollution. First, considering that winter heating is an important factor of haze pollution, the sample cities were divided into northern and southern cities for analysis. Columns (1) and (2) in Table 6 illustrate the regression results for the northern and southern subsamples. At the same time, the effect of smart city construction on haze pollution was more significant in the subsample of southern cities compared to northern cities. This may be because heating in northern areas consumes a large amount of coal-based energy, and the application of next-generation information technology in heating systems has not yet significantly reduced the corresponding particulate emissions. Second, the sample was divided into two subsamples: coastal cities and inland cities, considering the differences among cities in terms of climate and openness level. The estimated results are shown in columns (3) and (4) of Table 6, illustrating that the inhibitory effect of the smart city pilot policy on haze pollution is mainly reflected in inland cities, and the effect is not significant in coastal cities. This may be because coastal cities have mild climates and abundant precipitation, and their natural conditions are more conducive to the diffusion of haze pollutants than those of inland cities. Second, the majority of coastal cities are more economically developed, and because of their deep participation in the division of labor in the global value chain, the level of green technology in their industrial development is higher than that of inland cities, such that there is limited space in policy to control haze through smart city pilot policies. Finally, considering the difference in resource efficiency of cities, the sample is divided into two sub-samples according to whether they are resource-efficient or not. Columns (5) and (6) of Table 6 show the estimated results, illustrating that the inhibitory effect of the smart city pilot policy on haze pollution is not significant in non-resource-efficient cities, but the effect reflected in resource-efficient cities is significant at 5% significance levels. This difference may be due to the fact that resource-efficient cities focus more on the efficient use of resources, and thus, their awareness of environmental protection is better than that of non-resource-efficient cities, so that the effects of implementing smart city pilot policies can be seen more quickly.

### 5.3. Other Benefit Analysis

To cope with the dual challenges of environmental pollution and climate change, China proposed the collaborative governance idea of “pollution reduction and carbon reduction”. Therefore, this study investigates whether the smart city construction has the same inhibiting effect on carbon emissions in addition to the haze governance effect; that is, whether the smart city pilot can realize the collaborative governance of haze pollution and carbon emissions. For this reason, this study adds carbon dioxide emissions and carbon intensity as explanatory variables for regression, and the regression results are shown in columns (1) and (2) of Table 7. Although the effect of smart city construction on carbon emissions is not significant, it has a significant inhibitory effect on carbon-emission intensity at the 5% significance level; that is, smart city construction significantly reduces the carbon-emission intensity of the pilot cities, indicating that the smart city pilot policy can achieve collaborative governance of haze pollution and carbon emissions to a certain extent. The above analysis found that smart city pilot policies can reduce haze pollution by increasing technological innovation and the intensity of environmental regulation. This extends to the question of whether the inhibitory effect of smart city construction on haze comes at the expense of economic growth? To examine this issue, the impact of smart city construction on the economic growth of pilot cities was further investigated by using the logarithm of real GDP of prefecture-level cities as the explanatory variable and using the dummy variable of smart city construction as the core explanatory variable, following the difference-in-difference method for empirical analysis. As shown in column (3) of Table 7, the smart city construction has a significant contribution to the GDP of the city, which indicates that the smart city pilot policy can successfully control haze and ensure economic growth.

## 6. Conclusions

Taking the smart city pilot policy as the entry point, this study assesses the haze governance effect of smart city construction using a multi-time DID method based on a panel of 216 prefecture-level cities across China from 2009 to 2018. The following results can be obtained: firstly, the smart city construction can significantly reduce the level of urban haze pollution. Specifically, it can achieve a 2.98% reduction in the annual average PM_2.5_ concentration. Secondly, the technical and environmental regulation effects are the main pathways in smart city construction for reducing the level of haze pollution. Thirdly, the impact of smart city construction on haze pollution levels is influenced by factors such as the geographical location of the city and resource endowment, and its haze reduction effect is more obvious in southern, inland cities and resource-efficient cities. Fourthly, smart city construction reduces haze pollution while reducing carbon emission intensity and promoting regional GDP, which demonstrates that smart city construction can not only achieve collaborative governance of haze and carbon emissions, but also considers economic growth at the same time.

The policy implications of this study are as follows. First, although the direct goal of smart city construction in China is to improve the level of urban governance and service capacity, green development in the form of intelligent environmental protection, energy-saving, and other forms throughout the entire process of smart city construction was also improved. The empirical results of this study also show that smart city construction can reduce the level of haze pollution in pilot cities; as such, improving the level of smartness can be an alternative path to reduce urban air pollution and improve the quality of healthy life. Second, smart city construction reduces the level of haze pollution by promoting technological innovation and improving the intensity of environmental regulation rather than adjusting the industrial structure. Thus, the future construction of smart cities should be optimized to adjust to the urban industrial system, to strengthen the construction and improvement of new industrial chains, support the development of new industries with high technology and low energy consumption, improve the degree of integration and application of new-generation information technology in the traditional industries of the city, and promote the transformation of industries into environmentally friendly ones. Third, the haze-reduction effect of smart cities is more significant in southern and inland cities; on the one hand, the smartness of heat and other energy systems remains to be improved, while on the other hand, the technological innovation level of industrial development in inland cities remains to be improved with the development of smart cities. Fourth, under the new round of technological revolution and industrial change, green development requires the construction of new smart cities not only based on digital intelligent technologies such as the Internet of Things and cloud computing, but also by adhering to focusing on the human perspective to promote the realization of its diversified goals, combined with the development of local advantages and characteristics to improve the quality of cities.

Based on the research in this paper, there are also some limitations, and the following aspects can be expanded. First, haze pollution shows obvious cyclical characteristics. In China, haze pollution is concentrated in a November–March outbreak, which makes PM_2.5_ have large variance in a year. This may have some influence on the model results. However, it is difficult to calculate the variance of PM_2.5_ concentrations due to the unavailability of daily mean or monthly mean PM_2.5_ concentration data from 2000–2018. This issue will be further investigated if higher-frequency data are available. Second, haze pollution is spatially metastatic and mobile, so spatial factors can be further incorporated in the future to select a suitable method to analyze the spatial effects of smart-city pilot policy implementation in the mechanism analysis section. Third, in addition to the smart city pilots in this paper, the construction of emerging smart cities has been proposed in recent years, and the policy effects of their construction can be evaluated in future studies. Finally, this paper measures the overall effect of the pilot construction of smart cities on haze management. This may be considered further by studying the threshold effect of the different level of urban smartness on haze reduction, exploring the specific threshold values that produce the effect during policy implementation.

## Figures and Tables

**Table 1:** Descriptive statistics of variables.

Variable Name	Variable Symbols	Observations	Average Value	Standard Deviation	Minimum Value	Maximum Value
Haze Pollution Level	PM_2.5_	2160	42.535	19.991	3.596	110.121
CO_2_ Emissions	lnCO_2_	1944	2.924	0.707	0.860	4.687
CO_2_ Emission Intensity	CI	1944	2.285	1.402	0.470	10.757
Smart City Pilot	Smart	2160	0.258	0.438	0.000	1.000
Economic Development Level	lnPgdp	2137	10.347	0.676	8.353	12.655
	(lnPgdp)^2^	2137	107.511	14.169	69.78	160.143
Climatic Condition	lnRain	2150	9.110	0.487	7.520	10.220
Government Size	lnFis	2160	2.917	0.435	1.863	4.632
Level of Financial Development	lnFin	2159	4.354	0.453	2.468	6.613
Greening Level	lnGreen	2105	2.174	1.007	−2.496	5.293
Level of External Opening	lnFdi	2015	−0.184	1.351	−8.333	3.045

**Table 2:** Basic estimation results.

Explanatory Variables	Explained Variables: PM_2.5_	
	(1)	(2)
Smart City Pilot	−1.153 **	−1.270 **
	(−2.452)	(−2.520)
Actual GDP Per Capita		−35.677 ***
		(−3.979)
Squared Term of Actual GDP Per Capita		1.283 ***
		(3.000)
Climatic Condition		−4.077 ***
		(−3.747)
Government Size		2.213 *
		(1.833)
Level of Financial Development		2.216 ***
		(3.155)
Greening Level		0.724 *
		(1.696)
Level of External Opening		−0.674 ***
		(−3.988)
Constant	42.833 ***	294.850 ***
	(253.133)	(5.795)
City fixed effect	Yes	Yes
Year fixed effect	Yes	Yes
Observations	2160	1955
R^2^	0.933	0.938

Note: t-statistics in parentheses, ***, **, and * indicate 1%, 5%, and 10% significance levels, respectively.

**Table 3:** Regression results for the self-selection problem and considering other policies.

	Add Benchmark Variables	Other Environmental Policies
	(1)	(2)
Smart City Pilot	−1.0954 *	−1.2518 **
	(−1.829)	(−2.482)
Low Carbon City Pilot		0.7120
		(1.136)
Special Emission Limit Value Policy for Air Pollutants		−2.0817 **
		(−2.401)
Air Pollution Prevention and Control Action Plan		0.4150
		(0.720)
Control variables	Yes	Yes
City fixed effect	Yes	Yes
Year fixed effect	Yes	Yes
Observations	1599	1955
R^2^	0.940	0.938

Note: t-statistics in parentheses, ** and * indicate 5% and 10% significance levels, respectively.

**Table 4:** Regression results of the robustness test.

Explanatory Variables	Explained Variables: PM_2.5_			
	(1)	(2)	(3)	(4)
Smart City Pilot	−1.299 ***	−1.443 ***	−1.281 **	−0.8985
	(−2.599)	(−3.204)	(−2.444)	(−1.465)
Actual GDP Per Capita	−35.000 ***	−31.682 ***	−62.914 ***	−32.8034 ***
	(−4.012)	(−3.954)	(−6.202)	(−3.696)
Squared Term of Actual GDP Per Capita	1.286 ***	1.176 ***	2.794 ***	1.1403 ***
	(3.090)	(3.077)	(5.788)	(2.697)
Climatic Condition	−4.009 ***	−4.033 ***	−7.567 ***	−4.1248 ***
	(−3.735)	(−4.149)	(−6.671)	(−3.786)
Government Size	2.834 **	2.679 **	4.388 ***	2.3750 **
	(2.387)	(2.483)	(3.263)	(1.969)
Level of Financial Development	2.481 ***	2.141 ***	0.050	2.1559 ***
	(3.606)	(3.411)	(0.068)	(3.059)
Greening Level	0.799 *	0.707 *	0.386	0.7386 *
	(1.917)	(1.855)	(0.869)	(1.725)
Level of External Opening	−0.626 ***	−0.527 ***	−0.789 ***	−0.6919 ***
	(−3.752)	(−3.486)	(−4.129)	(−4.093)
Constant	283.052 ***	263.393 ***	449.599 ***	280.6227 ***
	(5.707)	(5.793)	(7.832)	(5.558)
City fixed effect	Yes	Yes	Yes	Yes
Year fixed effect	Yes	Yes	Yes	Yes
Observations	1867	1955	1764	1955
R^2^	0.936	0.944	0.939	0.938

Note: t-statistics in parentheses, ***, **, and * indicate 1%, 5%, and 10% significance levels, respectively.

**Table 5:** Test results of mechanism analysis.

	Benchmark Results	Conduction Mechanisms					
		Technological Innovation		Industrial Structure		Environmental Regulation	
	(1)	(2)	(3)	(4)	(5)	(6)	(7)
Smart City Pilot	−1.270 **	0.060 ***	−1.113 **	0.485 *	−1.260 **	−6.113 ***	−1.203 **
	(−2.520)	(3.023)	(−2.213)	(1.749)	(−2.496)	(−3.414)	(−2.360)
Mechanism Variable			−3.047 ***		0.002		0.033 ***
			(−4.972)		(0.055)		(4.779)
Actual GDP Per	−35.677 ***	−2.831 ***	−44.011 ***	59.613 ***	−35.777 ***	204.316 ***	−40.255 ***
Capita	(−3.979)	(−8.088)	(−4.848)	(12.095)	(−3.831)	(6.259)	(−4.298)
Squared Term of Actual GDP Per Capita	1.283 ***	0.137 ***	1.683 ***	−2.194 ***	1.288 ***	−8.730 ***	1.530 ***
	(3.000)	(8.227)	(3.884)	(−9.333)	(2.938)	(−5.629)	(3.446)
Climatic Condition	−4.077 ***	0.149 ***	−3.713 ***	0.601	−4.072 ***	−4.137	−4.188 ***
	(−3.747)	(3.509)	(−3.420)	(1.005)	(−3.741)	(−1.071)	(−3.820)
Government Size	2.213 *	−0.276 ***	1.304	−7.191 ***	2.162 *	−6.081	1.242
	(1.833)	(−5.867)	(1.076)	(−10.824)	(1.731)	(−1.397)	(1.006)
Level of Financial Development	2.216 ***	0.009	2.267 ***	−2.289 ***	2.251 ***	−0.853	3.094 ***
	(3.155)	(0.316)	(3.248)	(−5.924)	(3.169)	(−0.328)	(4.204)
Greening Level	0.724 *	−0.038 **	0.607	−0.114	0.659	−1.869	0.710 *
	(1.696)	(−2.299)	(1.430)	(−0.483)	(1.532)	(−1.243)	(1.664)
Level of External	−0.674 ***	0.022 ***	−0.603 ***	0.019	−0.679 ***	−1.143 *	−0.647 ***
Opening	(−3.988)	(3.331)	(−3.576)	(0.201)	(−4.016)	(−1.888)	(−3.766)
Constant	294.850 ***	14.308 ***	338.303 ***	−308.103 ***	295.320 ***	−1069.072 ***	314.544 ***
	(5.795)	(7.206)	(6.593)	(−11.015)	(5.609)	(−5.774)	(5.932)
City fixed effect	Yes	Yes	Yes	Yes	Yes	Yes	Yes
Year fixed effect	Yes	Yes	Yes	Yes	Yes	Yes	Yes
Observations	1955	1952	1952	1954	1954	1897	1897
R^2^	0.938	0.751	0.939	0.924	0.938	0.807	0.940

Note: t-statistics in parentheses, ***, **, and * indicate 1%, 5%, and 10% significance levels, respectively.

**Table 6:** Results of heterogeneity analysis.

	Northern Cities	Southern Cities	Coastal Cities	Inland Cities	Resource-Efficient Cities	Non-Resource-Efficient Cities
	(1)	(2)	(3)	(4)	(5)	(6)
Smart City Pilot	−1.219	−1.851 ***	−1.042	−1.132 **	−1.939 **	−0.756
	(−1.601)	(−3.024)	(−1.144)	(−1.991)	(−2.394)	(−1.190)
Actual GDP Per Capita	−1.578	−41.397 ***	19.259	−39.471 ***	−14.859	−53.154 ***
	(−0.102)	(−4.177)	(1.159)	(−3.749)	(−0.999)	(−4.763)
Squared Term of Actual GDP Per Capita	−0.305	1.660 ***	−1.478 *	1.483 ***	0.538	1.997 ***
	(−0.423)	(3.462)	(−1.842)	(2.955)	(0.761)	(3.700)
Climatic Condition	−7.269 ***	4.015 ***	−5.360 ***	−2.725 **	−3.753 **	−4.273 ***
	(−4.446)	(2.709)	(−3.499)	(−2.085)	(−2.144)	(−3.118)
Government Size	1.789	2.396 *	−8.957 ***	3.628 ***	0.116	4.080 **
	(0.901)	(1.717)	(−3.981)	(2.693)	(0.064)	(2.517)
Level of Financial Development	2.192 **	2.457 **	−1.675	2.920 ***	3.556 ***	0.968
	(2.284)	(2.412)	(−1.491)	(3.632)	(3.242)	(1.064)
Greening Level	0.484	1.093 **	−0.278	0.693	0.306	1.041 *
	(0.734)	(2.171)	(−0.392)	(1.434)	(0.512)	(1.689)
Level of External Opening	−0.782 ***	−0.762 ***	−0.913 ***	−0.542 ***	−0.661 ***	−0.824 ***
	(−3.199)	(−3.500)	(−2.839)	(−2.862)	(−2.734)	(−3.472)
Constant	144.023	233.026 ***	82.515	293.759 ***	154.095 *	403.469 ***
	(1.621)	(4.093)	(0.861)	(4.923)	(1.868)	(6.263)
City fixed effect	Yes	Yes	Yes	Yes	Yes	Yes
Year fixed effect	Yes	Yes	Yes	Yes	Yes	Yes
Observations	922	1033	337	1618	824	1131
R^2^	0.950	0.929	0.957	0.937	0.936	0.940

Note: t-statistics in parentheses, ***, **, and * indicate 1%, 5%, and 10% significance levels, respectively.

**Table 7:** Other benefits regression results.

	Logarithm of CO_2_ Emissions	Carbon Dioxide Emission Intensity	Logarithm of Real GDP
	(1)	(2)	(3)
Smart City Pilot	−0.0002	−0.052 **	0.012 ***
	(−0.037)	(−2.343)	(2.646)
Climatic Condition	0.038 ***	0.080	0.045 ***
	(2.946)	(1.633)	(4.665)
Government Size	−0.027 *	0.291 ***	−0.158 ***
	(−1.808)	(5.167)	(−15.192)
Level of Financial Development	0.023 ***	0.076 **	−0.047 ***
	(2.791)	(2.413)	(−7.608)
Greening Level	−0.007	−0.119 ***	0.016 ***
	(−1.337)	(−6.273)	(4.318)
Level of External Opening	−0.005 **	−0.040 ***	0.014 ***
	(−2.170)	(−4.834)	(9.364)
Constant	2.602 ***	0.510	7.169 ***
	(19.536)	(1.010)	(73.270)
City fixed effect	Yes	Yes	Yes
Year fixed effect	Yes	Yes	Yes
Observations	1764	1764	1955
R^2^	0.993	0.963	0.996

Note: t-statistics in parentheses, ***, **, and * indicate 1%, 5%, and 10% significance levels, respectively.

## Data Availability

Publicly available datasets were analyzed in this study. PM_2.5_ data were collected from the Atmospheric Composition Analysis Group of Dalhousie University in Canada; the data of CO_2_ emission and CO_2_ emission intensity come from the study of Chen et al.; the smart city pilot list announced by the Ministry of Housing and Construction; the data of control variables were obtained from the China City Statistical Yearbook and the China Regional Statistical Yearbook.
